# Supplementary material for: Pneumococcal H₂O₂ reshapes mitochondrial function and reprograms host cell metabolism
Source: mBio. 2025 Oct 31;16(12):e02019-25. doi: 10.1128/mbio.02019-25 (PMC12691681; doi:10.1128/mbio.02019-25)
Supplement: Supplemental Figures — Figures S1-S5. [file mbio.02019-25-s0001.pdf]

Supplemental Figures

Fig. S1.

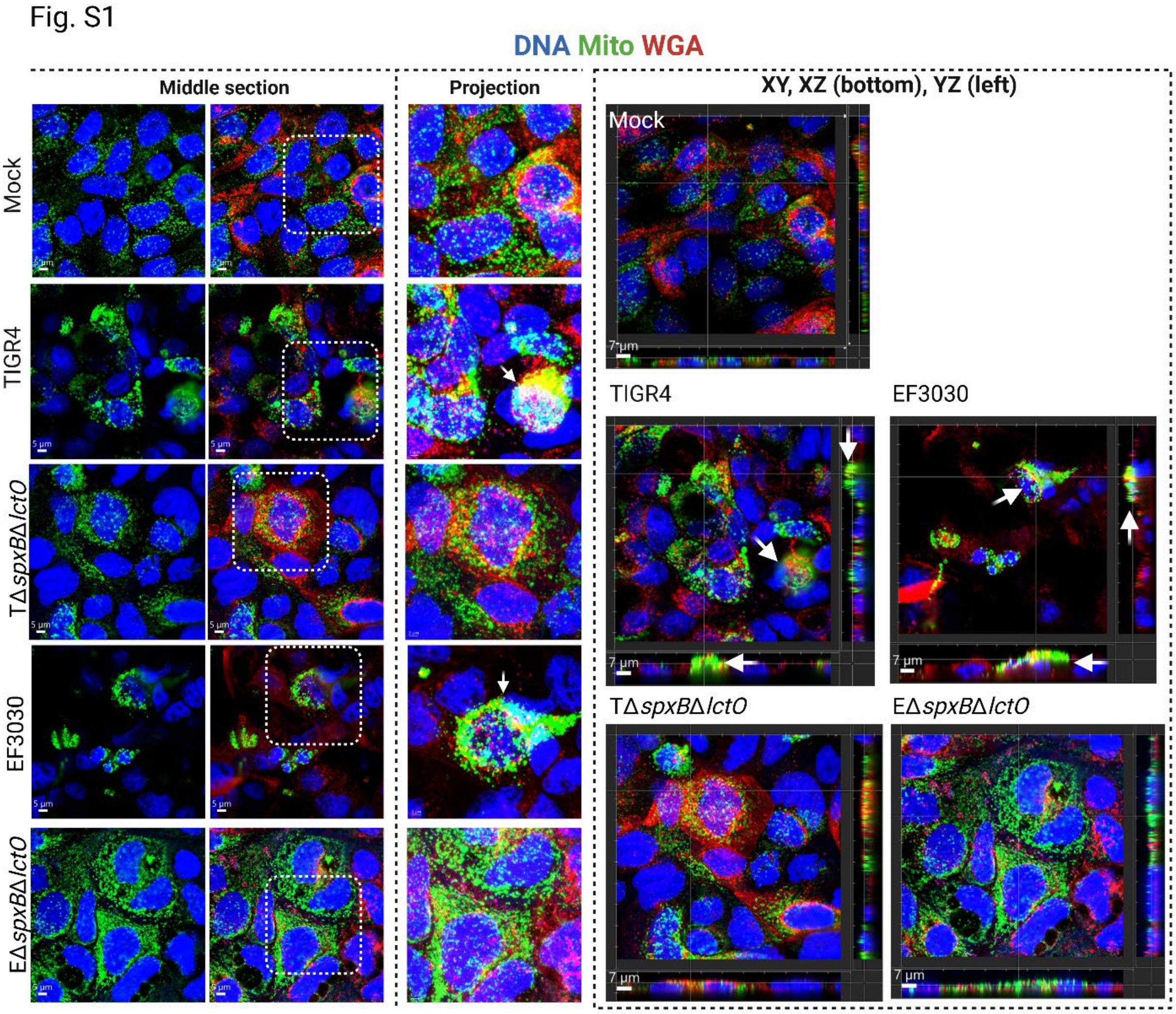

Figure S1. Spn-H_2O_2 induces mitochondrial morphological changes and aggregation in human bronchial Calu-3 cells. Cells were either mock-infected or infected with Spn strains and incubated for 8 h at 37°C with 5% CO_2. Infected cells were stained for mitochondria (green), DNA (blue), and cell membrane (WGA, red), then imaged using confocal microscopy, and analyzed with Imaris software. Panels (left) depict XY optical-middle sections (0.5 µm), (middle) projections or (right) XY, XZ (bottom), and YZ (side) optical sections derived from z-stacks, as indicated. The

dotted square in middle sections denotes the area selected for projection generation. Arrows highlight regions of mitochondrial aggregation. Images are representative of two independent experiments.

Fig. S2.

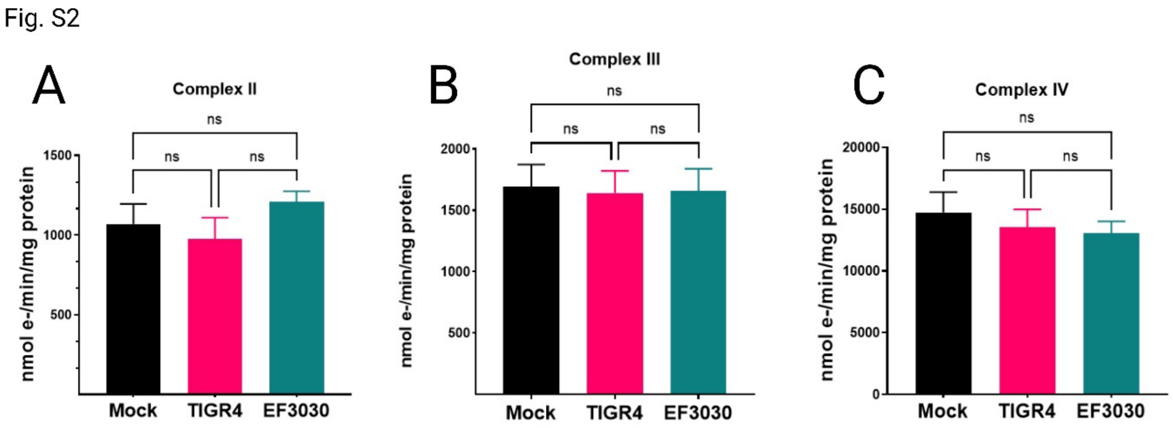

Figure S2. Spn-H_2O_2.does not affect oxidative capacity of mitochondrial complexes II, III or IV. Heart mitochondria were treated with filter-sterilized *Streptococcus pneumoniae* (Spn) culture supernatants collected after 4 h of growth. Oxidative capacity of complex II, III and IV activity was measured as the time-dependent oxidation of 2,6-dichloroindophenol sodium salt hydrate (DCPIP), reduction of cytochrome c, and oxidation of N,N,N',N'-Tetramethyl-p-phenylenediamine dihydrochloride (TMPD), respectively. Data represent mean ± SEM, one-way ANOVA with Dunnett's post hoc test, ns, not significant.

Fig. S3

Fig. S3

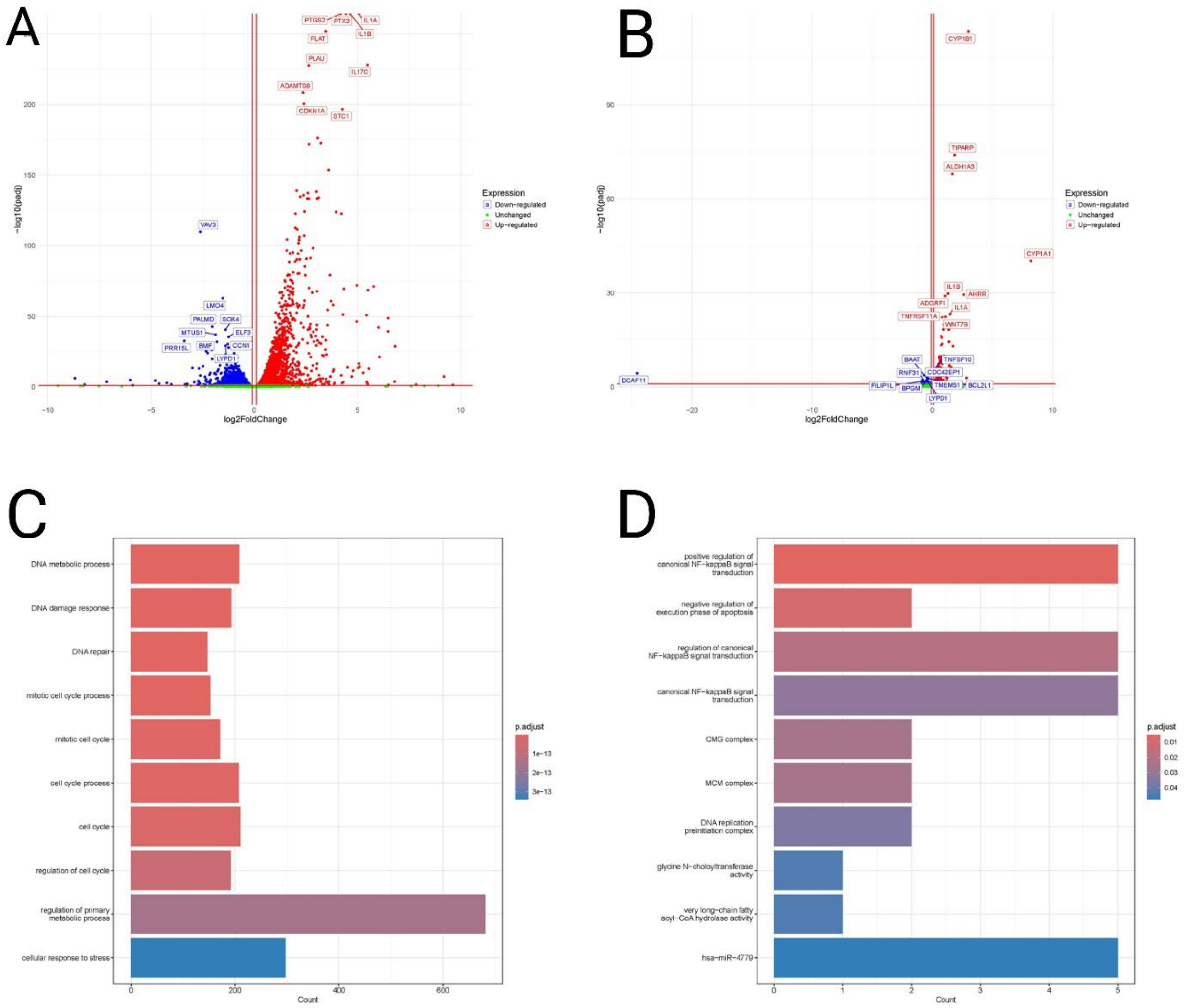

Figure S3. Transcriptional reprogramming in calu-3 cells infected with Spn strains. Calu-3 cells were infected with *Streptococcus pneumoniae* (Spn) strains TIGR4 (A, C) or its isogenic Δ*spxB* mutant (B, D) for 4 h. (A, B) Volcano plots display differential gene expression ($\log_2$ fold change vs. -$\log_{10}$ FDR), with downregulated (blue), upregulated (red), and unchanged (gray) genes. (C, D) Bar graphs show Gene Ontology (GO) enrichment of significantly altered pathways, with adjusted p-values indicated by color gradient. Data represent mean values from two biological replicates with technical triplicates; statistical details are in the methods section.

Fig. S4.

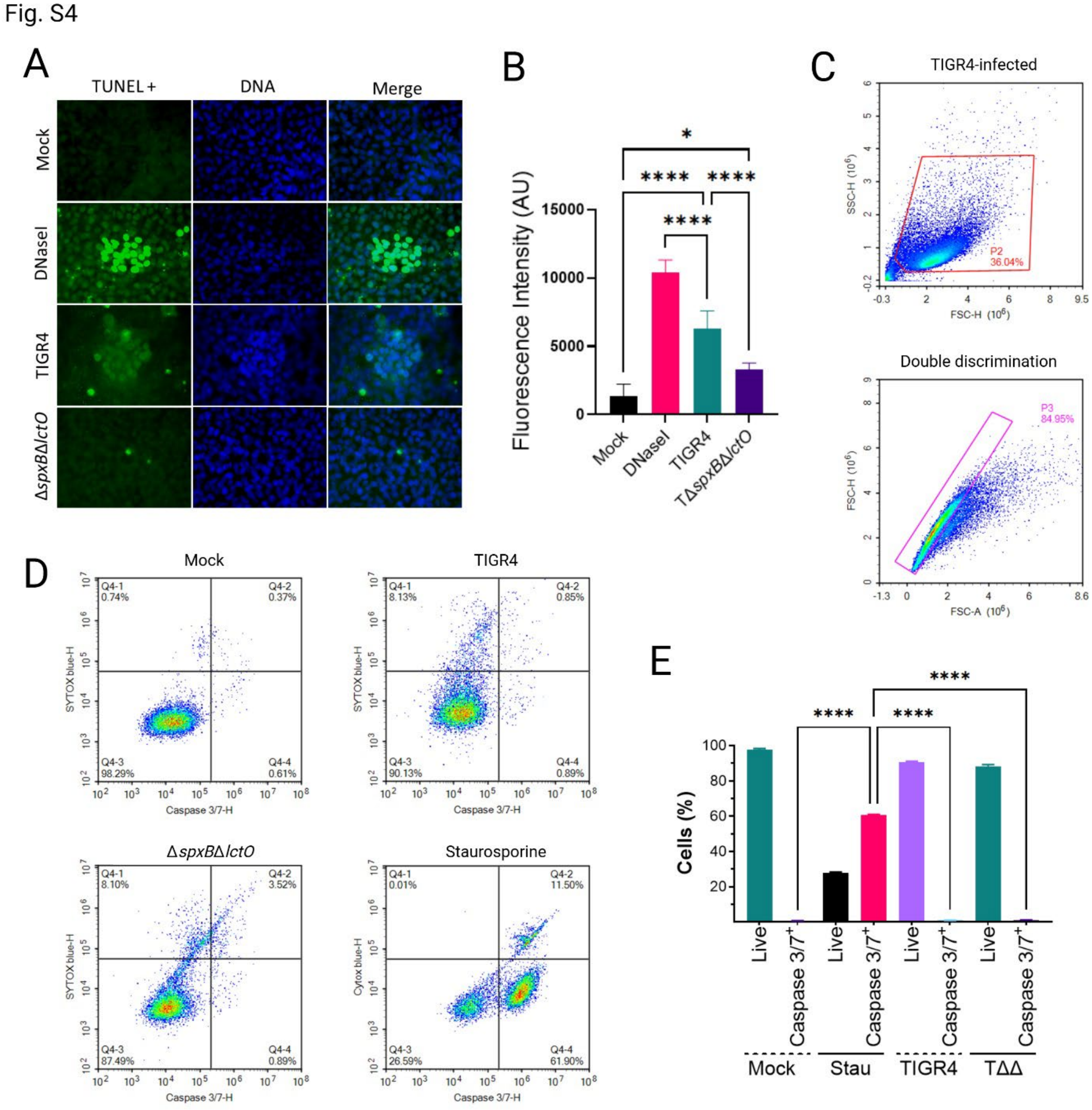

Figure S4. Analysis of apoptosis and cell death in A549 cells under various conditions. (A) Human alveolar A549 cells were mock-infected, treated with DNase I (1 U), or infected with *Streptococcus pneumoniae* strains TIGR4 or TIGR4ΔspxBΔlctO for

10 h. Cells were stained using the Click-iT Plus TUNEL assay, mounted with DAPI-containing resin, and analyzed by confocal microscopy with Imaris software. (B) Quantification of TUNEL staining, reported as fluorescence intensity (FITC/GFP) in arbitrary units (AU) for each condition. Data represent mean ± SEM from two independent experiments with four internal replicates each; one-way ANOVA with Dunnett's post hoc test; $p<0.05$, *$p<0.0001$. (C–D) A549 cells were mock-infected, infected with TIGR4 or TIGR4ΔspxBΔlctO for 10 h, or treated with staurosporine (10 µM, 4 h). Cells were stained with CellEvent Caspase-3/7 Green Detection Reagent (2 µM) and SYTOX Blue Dead Cell Stain (1 µM) and analyzed by flow cytometry. (D) Representative bivariate density plots of 10,000 single cells (gated in P3, normalized across samples) showing non-apoptotic SYTOX Blue–positive cells (Q4-1), SYTOX Blue–negative non-apoptotic cells (Q4-3), and apoptotic caspase-3/7–positive, SYTOX Blue–negative cells (Q4-4). (E) Percentage of SYTOX Blue–negative live cells and the proportion of caspase-3/7–positive cells among them. Data represent mean ± SEM from two independent experiments with two internal replicates each; one-way ANOVA with Dunnett's post hoc test; ***$p<0.0001$.

Fig. S5.

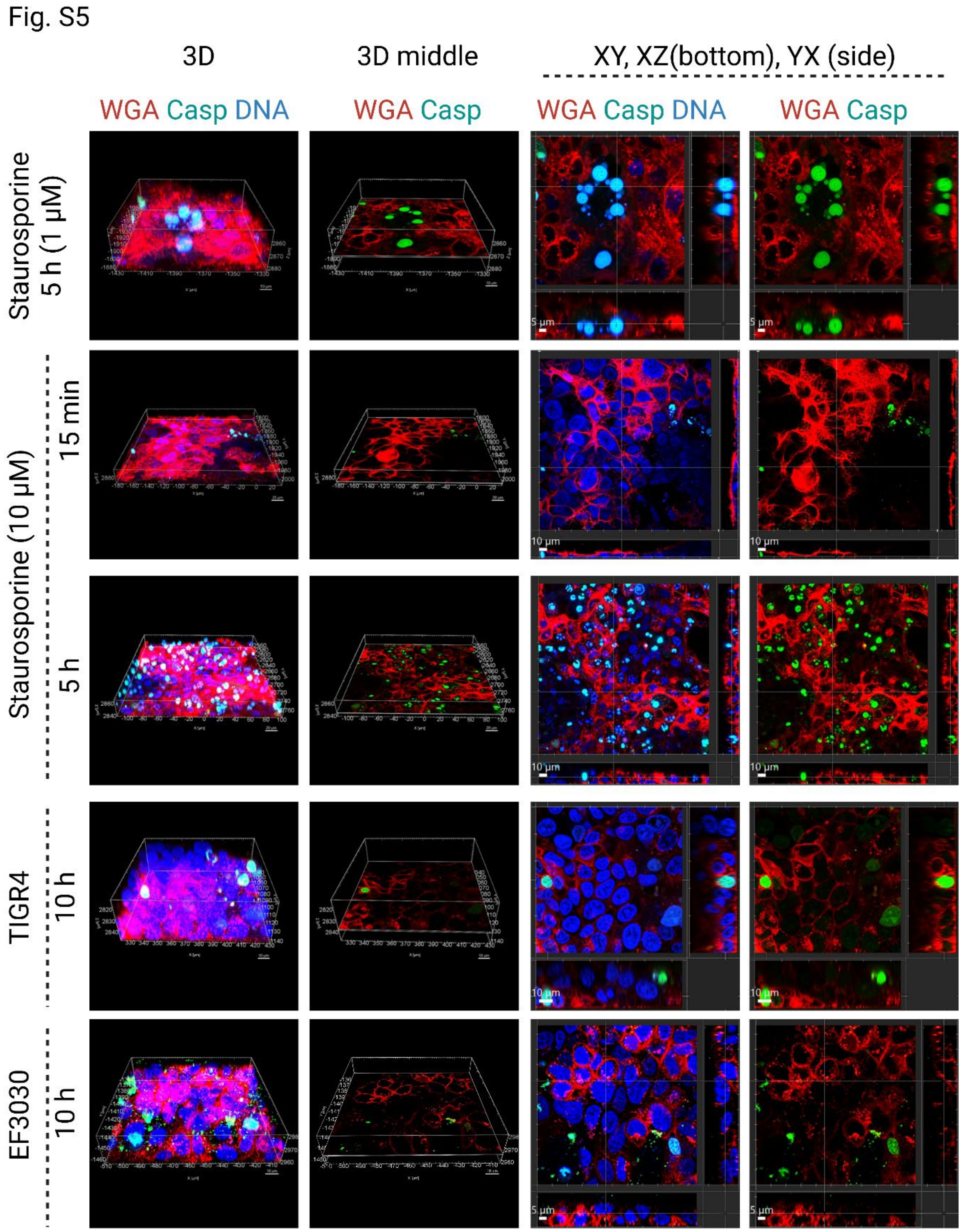

Fig. S5
3D
3D middle
XY, XZ(bottom), YX (side)
WGA Casp DNA
WGA Casp
WGA Casp DNA
WGA Casp
Staurosporine 5 h (1 µM)
Staurosporine (10 µM)
15 min
5 h
TIGR4
10 h
EF3030
10 h
5 µm
10 µm

Figure S5. Limited Contribution of *Streptococcus pneumoniae*-Derived H_2O_2 to Apoptosis in Lung Epithelial Cells. (A) Human alveolar epithelial A549 cells were treated with staurosporine (1 μM or 10 μM) for 5 h or infected with S. pneumoniae strains TIGR4 or EF3030 for 10 h. At the time of infection, cells were stained with wheat germ agglutinin (WGA) for membrane labeling, CellEvent Caspase-3/7 Green Detection Reagent (Casp) for apoptosis detection, and DAPI for nuclear visualization. Z-stacks were acquired in real-time using confocal microscopy at the indicated time points. Shown are 3D reconstructions (left panels) and mid-plane sections of z-stacks (middle panels), alongside corresponding XY, XZ, and YZ optical sections (right panels). Scale bars are indicated where applicable.
