# Supplementary material for: Pneumococcal H₂O₂ reshapes mitochondrial function and reprograms host cell metabolism
Source: mBio. 2025 Oct 31;16(12):e02019-25. doi: 10.1128/mbio.02019-25 (PMC12691681; doi:10.1128/mbio.02019-25)
Supplement: Legend — for Video S1. [file mbio.02019-25-s0002.docx]

Video S1. Time-lapse imaging of caspase-3/7 activation in Calu-3 cells during staurosporine treatment and TIGR4 infection.

Time-lapse confocal microscopy was performed on Calu-3 cells treated with staurosporine (10 µM) for 7 h or infected with Streptococcus pneumoniae strain TIGR4 for 10 h. Cells were stained with CellEvent Caspase-3/7 Green Detection Reagent (2 µM) to monitor caspase-3/7 activation, with wheat germ agglutinin (WGA) for membrane labeling, and DAPI for nuclear visualization. Imaging was conducted every 2.5 minutes over the indicated time courses using a confocal microscope and analyzed with Imaris software. Staurosporine-treated cells exhibit robust caspase-3/7 activation (green fluorescence) beginning at 4 h post-incubation, indicative of apoptosis, while TIGR4-infected cells show minimal caspase-3/7, consistent with limited apoptosis and predominant cell death via non-apoptotic pathways. Scale bar: 5 µm. Data are representative of two independent experiments.
